# Impact of permagarden intervention on improving fruit and vegetable intake among vulnerable groups in an urban setting of Ethiopia: A quasi-experimental study

**DOI:** 10.1371/journal.pone.0213705

**Published:** 2019-12-02

**Authors:** Fikralem Alemu, Medhanit Mecha, Girmay Medhin

**Affiliations:** 1 Fikralem Alemu, Ethiopian Institute of Water Resources, Ethiopia, Addis Ababa; 2 Medhanit Mecha, Family Health International Ethiopia, Ethiopia, Addis Ababa; 3 Girmay Medhin, Aklilu Lemma Institute of Pathobiology, Addis Ababa University, Addis Ababa; Baylor University, UNITED STATES

## Abstract

**Background:**

Increasing nutrient intake through home gardening is a sustainable way to address multiple micronutrient deficiencies in developing countries. This study investigated the impact of permagarden intervention in increasing the frequency and diversity of vegetable and fruit consumption among vulnerable families in seven cities of Ethiopia.

**Method:**

A quasi-experimental study was conducted from August 10 to September 30, 2015. A total of 884 care givers (427 from intervention and 457 from control) participated in the study. Data were collected through face to face interviews with caregivers of highly vulnerable children. Propensity score matching (PSM) was used as implemented in STATA software. Program impact on the frequency and diversity of households’ fruit and vegetable consumption between intervention and control groups was assessed using chi square test.

**Results:**

Intervention participants had a 13% higher increase in frequency of vegetable and fruit consumption compared with control participants (p<0.01). Diversity (consumption of 2 or more groups of vegetable and fruit) is higher among intervention groups than control groups (percentage difference = 9, p-value<0.05). A significant higher percentage of participants in intervention group reported getting the one-week vegetable and fruit mainly from their own garden (percentage difference 58.3%, p<0.05). A significantly larger proportion of participants in the intervention group compared to control group reported “high likelihood” on intention to grow vegetables in the future (percentage difference = 30%, and P<0.01). Perceived importance to include vegetable in everyday meal was higher among intervention group participants than control group participants (percentage difference = 11.5%, P<0.01).

**Conclusions:**

The observed higher FV intake among permagarden intervention group compared to control group suggest that nutrition and health programs need to promote permagarden as a means to improve FV intake among vulnerable societies in resource limited countries.

## Introduction

Despite a growing body of evidence that suggests eating fruit and vegetable (FV) prevents several diseases, low consumption of FV remains one of the top 10 global risk factors for mortality [1,2]. More than 2.7 million lives could be saved annually by increasing individual FV consumption [1]. The World Health Organization (WHO) defined low FV consumption of an individual as consuming less than the recommended intake of at least 400g of fruits and/or vegetable a day which is equivalent to five servings [3,4]. In developing countries fruit and vegetable provide 80% of vitamin A dietary supply [5].

Undernutrition continues to account for 21% of the global disease burden among children in low and middle income countries [6]. It is the major contributor of diarrheal morbidity among children less than five years of age, with a vicious cycle existing between diarrhea and under-nutrition [7,8]. More than 30% (163 million) young children in developing countries are affected by vitamin A deficiency (VAD) [9]. VAD is responsible for 6% of deaths in Africa [10]

With an estimated population of 104 million in 2018, Ethiopia is one of the largest least developed countries (LDCs) in Sub-Saharan Africa [11]. About 10.2 million Ethiopians experienced food insecurity in 2016 [12]. Ethiopia has more than 5 million orphans and vulnerable children [13] that could be prone to food insecurity [14]. More than 51% of the deaths in under-five children are attributed to undernutrition [15]. According to the National Demographic Health Survey (DHS) 2016 report, an estimated 38% of children less than five years of age were stunted, 10% were wasted, and 24% were underweight [16].

FV from home gardens have been documented as important supplemental sources of nutrients, contribute to food and nutritional security, and allow families to produce their own FV organically [17,18,19]. Vegetable gardens introduce more variety into the diets of children [20]. Consequently, the intake of various nutrients such as vitamin A and calcium is increased with gardening [21]. Home vegetable gardening also has psychosocial benefits. Learning gardening skills brings a sense of personal satisfaction to gardeners [22], and community gardens provide an opportunity to socialize with other community members beyond supplementing daily household consumption [23]. The above-mentioned studies contribute towards an improved understanding of the benefits of home gardens that include access to food and nutrition, social and psychological benefits. However, most of these studies were conducted in developed countries [24,25]. The impact of home gardens in developing countries is not well documented.

In the last decade, home garden intervention efforts in Ethiopia have been less successful. A gardening intervention evaluation that was conducted after 5 years of implementation (2006–2011) in Ethiopia reported that most school gardens failed to sustain because of several factors that include lack of ownership by the target groups, inability to maintain technologies such as drip irrigation, inconvenience due to long distance from home, and lack of control of gardens by individual gardeners [26]. Combining the best methods of permaculture with those of bio-Intensive gardening, permagarden is a home gardening techniques which applies only locally available materials, tools, seeds and plants [27]. Permagarden technique produces high yields from very small plots of land with a relatively small amount of water and low investment in labor. However, there is limited evidence on the impact of permagarden interventions in improving FV consumption among vulnerable groups in urban settings of low- and middle-income countries.

Yekokeb Berhan (YB) (2011–2017) was a USAID- funded program that aimed to reduce the vulnerability of Highly Vulnerable Children (HVC) and their families in Ethiopia. The program was designed to strengthen systems and structures to deliver quality essential services to HVC and their families that include health, education, economic strengthening, psychosocial support, food and nutrition and legal protection. According to the YB program, HVC are those children under the age of 18 years whose safety, well-being or development is at significant risk due to inadequate care, protection or access to essential services [28]. A standard tool called Child Support Index (CSI) was used to assess children’s status in terms of their health, nutrition, psychosocial, protection, shelter, and care [29]. Children with lowest CSI score were enrolled in the program. With Pact, a prime grant recipient organization technical support from FHI 360 and Child Fund, the program was implemented by 49 local Civil Society Organization (CSOs) as well as with the Government of Ethiopia [30].

Permagarden is one of the interventions in the YB program that was implemented to address the food and nutrition needs of HVC and their families [30]. The current study aimed to answer the question “Has permagarden intervention resulted in increased frequency and diversity of vegetable consumption?

## Material and methods

### Study design and setting

A quasi-experimental study design that involved intervention and control groups without baseline data was employed. Administratively, Ethiopia is structured into nine regional federal states and two city administrations. Each region is sub-divided into a set of zones, each zone is divided into set of woreda (an equivalent of a district) and each woreda is divided into kebeles. A *kebele* is the lowest government administrative unit [31]. The study was conducted in 7 districts of two regions of Ethiopia, Amhara (5 districts) and Tigray (2 districts) where YB program was implemented. The 2016 DHS report shows that stunting, underweight, and wasting prevalence are higher in these two regions compared to the other regions, or compared to the national prevalence [32]. The study included neighboring woreda that have almost similar socio-economic characteristics with the intervention areas as a control. Controls are caregivers that were enrolled into the YB program and they were getting integrated health, education, child protection, psychosocial services but they were not targeted for permagarden intervention due to limited capacity.

### Description of the intervention

Permagarden intervention was home gardening that was focused on training in permagarden skills, provision of gardening tools and seeds. The main objective of this intervention was to create access to fresh FV for families of highly vulnerable children. A five-day long Training of Trainers (ToT) on permagardening was provided for community facilitators who were responsible to train, rollout and follow up the permagarden implementation. A group of 8 to 12 caregivers and volunteers were trained for 3 days by the trained community facilitators at gardens of caregivers’ house or at community centers close to the caregivers’ house. A visual and out of class room practical training approach was used in which all trainees practiced every step of garden establishment. The key training topics include local resource identification, compost preparation, water capture and control, bio-intensive garden bed creation and planting, making natural pest controls, overall garden management throughout the year including crop rotation, and nutritional balanced diet. From April 2013 to May 2015, 17,500 caregivers were trained in the 9 regions of Ethiopia. Trained volunteers and community facilitators visited caregivers’ garden once every two weeks and provided technical support.

### Sample size estimation and sampling procedure

Sample size estimation was based on a statistical approach for calculating sample size for population proportion for intervention and control group. Sample size was calculated assuming an increase of 10 percentage points in proportion of households exhibiting FV consumption compared with the control groups as a result of the intervention. In the control groups it was assumed that 50 percent of households consume FV with 95% level of significance and 80% statistical power, the estimated sample size was 388 for each. An additional 10% was added to this sample size to account for possible non-response and matching failures making the total sample size 427 caregivers in each of the two arms.

We selected five *woreda* from Amhara region and two *woreda* from Tigray regions using simple random sampling technique from a list of intervention woredas. A total of 1525 caregivers were engaged in permagarden intervention in seven selected study woredas. Study participants were proportionally allocated into seven woreda of the two regions (309 in Amhara region and 118 in Tigray region), and they were selected using simple random sampling technique. Inclusion criteria for selection of intervention group were: being a caregiver of at least one highly vulnerable and under the age of 18 child, being enrolled in YB program two years prior to the study period, targeted by permagarden intervention (trained and provided technical support) and having a very small piece of land in their yard starting from 2 meters by 2 meters. The inclusion criteria for the control group include: being a resident in the study area for at least one year, being a caregiver of HVC, not ever targeted by gardening intervention, and having a small piece of land starting from 2 meters by 2 meters in their yard. Caregivers that were not physically well were not included in the current study.

### Data collection methods and measurements

Home garden is a common intervention that aims to increase consumption of vitamin A-rich [33]. The difference in a 24 hour diversity of vitamin A-rich FV consumption and a one-week frequency of any FV intake between intervention and control groups was measured to assess whether there is an increase in overall FV consumption as a result of permagarden intervention. The Food and Agriculture Organization (FAO) of the United Nations has developed a list of 12 food groups that a household can consume over the preceding 24 hour for measuring household dietary diversity [34]. Questions were adopted from a list of 12 food groups in FAO guideline [34] to assess vitamin A rich FV consumption. The FV groups are: group I (plant foods rich in vitamin A and tubers such as pumpkin, carrot, squash, or sweet potato, etc.), group II (plant foods rich in vitamin A fruits (ripe mango, cantaloupe, apricot, ripe papaya, etc.), group III (dark green leafy vegetables, including cassava leaves, kale, spinach, etc.) and group IV (any other vegetables e.g. tomato, onion, eggplant or other locally available vegetables). The person who was responsible for meal preparation for the household the previous day were asked about vitamin A rich fruits and vegetables eaten inside the home during the previous day and night, by any member of the household. Households that reported consuming at least two FV groups were considered to have diversified FV consumption. The person who was responsible for meal preparation was asked about the frequency of one-week FV intake in the house by family members using a pre-coded response (never, once, twice to three times, four to six times, one and at least once in a day). Their response was further recategorized as not frequent (1 to 3 times), and frequent (greater than 3 times). Caregiver’s intention to practice home gardening in the future was assessed by using a precoded responses that range from 1 to 5, where 5 stands for extremely likely and 1 stands for not likely at all [35]. For reporting purposes response for intention were further recategorized in to less likely (scores 1 to 2), likely (score 3), and more likely (scores 4 to 5). The questionnaire also captured respondents background information that include age, gender, educational level, occupation, number of children that live in the house, ever trained on vegetable gardening, and household monthly income. The questionnaire was originally developed in English and translated into the local languages (Amharic and Tigrigna) by a certified local translator.

The data collection team consisted of two supervisors that have BSC degree and ten interviewers that have at least a high school education level who speak local language. All data collectors and supervisors were trained for 3 day on the study tools, interview skills and protection of human subjects through presentation, role play, discussion and field practice. The questionnaire was pre-tested with 50 households (25 intervention caregivers and 25 control caregivers) in two non-study neighborhood *woredas* (one in Amhara region and one in Tigray region). The study woreda were purposively selected for the pilot testing because of its administrative convenience. Participants for the pretest were randomly selected. During the pretest, questions were assessed for clarity and suitability to the participants, and adjustments were made based on findings. Face to face interviews were conducted using the final questionnaire. During the data collection, completed questionnaires were evaluated by the supervisors at the end of each day. Incomplete and inconsistent records were corrected in the field by re-visiting and re-examining study participants. In addition to administering the questionnaires, data collectors visited the respondents’ garden.

### Data management and data analysis

Cleaned data were double entered into Epidata software (EpiData Association, Odense, Denmark), and analyzed using STATA module psmatch2 developed by Leuven and Sianesi 2003 [36]. Propensity score matching with nearest neighbor matching algorithm was conducted on variables that were anticipated to influence outcomes including (a) ever trained on vegetable gardening by any organization, (b) household monthly income, (c) current participation in other gardening programs/intervention other than permagarden, (d) support from other source for vegetable gardening and participation in saving and lending groups. After balancing intervention and control groups with PSM, the program impact on the frequency and diversity of household’s FV consumption between intervention and control groups was assessed using chi-square and t-test. Percentage for categorical variables and mean values for continuous measurements were reported. Results were reported as being statistically significant whenever p-value was less than 5%.

### Ethical statement

The research proposal was reviewed and approved by FHI 360’s Protection of Human Subjects Committee, and The National Ethics Review Committee (NRERC). Permission to conduct the study was obtained from the Amhara and Tigray regional women, children and youth affairs bureaus. Participants were informed about the purpose, their role in the study, the potential benefits, and possible risks associated with participating in this research study. A written informed consent was obtained from all participants for the interview and for the publication.

## Results

### Socio-demographic characteristics of participants

Table 1 presents the socio-demographic characteristics of study participants. From the total of 884 households, 503 were from Amhara region (236 Intervention and 267 Control) and 381 were from Tigray region (191 Intervention and 190 Control). The mean age was 40.7 and 39.5 for the intervention and control group respectively, the difference was not statistically significant. Majority of respondents (90%) were women. About 40% from intervention and 50% from the control groups did not have formal education, the difference was statistically significant (P<0.001). About 58% caregivers in the control groups and 49.8% of the caregivers in the intervention group were not married p (P<0.05). One quarter (27.4% caregivers in the intervention groups and 21.7%) caregivers in control groups were self-employed. About 42% of the intervention group and 52% of the control group were daily laborers, the difference was statistically significant (p<0.05). Higher percentage of participants in intervention group reported they have participated in a saving group (69.8%) than in the control group (48.5%). There was also a significant difference in the proportion of respondents participating in small-scale business activities, with 38% in the intervention group compared to 19% in the control group. About 90% of households in the intervention and only 14% in the control groups were growing vegetable by the time of data collection (P<0.01).

**Table 1:** Socio-demographic characteristics of study participants (caregivers) in seven cities of Ethiopia (n = 852).

Households/Caregivers characteristics	Study group		Chi-Square test/ t-test	P-value
	Intervention (n = 427)	Control (n = 457)		
**Region (%)**				
Amhara	55.3	58.4	χ^2^ = 0.95	0.344
Tigray	46.7	41.6		
**Sex of caregiver (%)**				
Male	10.1	8.8	χ^2^ = 0.45	0.502
Female	89.9	91.2		
**Mean age of caregivers**	40.7	39.5	t = 1.51	
**Caregiver age group (%)**				
Less than 30	12	15.4	χ^2^ = 2.58	0.46
30–39	39.7	39.9		
40–49	26.1	25		
50 and above	22.2	19.7		
**Caregiver relation to HVC (%)**				
Mother	71.6	79.1	χ^2^ = 8.3	P<0.05
Father	26.1	18		
Others	2.3	2.9		
**Caregiver marital status (%)**				
Married/Cohabit	50.2	42.3	χ^2^ = 5.6	P<0.05
Single/Divorce/Widow	49.8	57.7		
**Caregiver level of Education (%)**				
No formal Educ.	40	50.6	χ^2^ = 20.8	P<0.001
Elementary 1–8	35.8	36.6		
Secondary & above	24.2	12.9		
**Caregiver occupation group (%)**				
Daily labor	42.2	52.3		
Gov. employee	6.8	3.2	χ^2^ = 12.1	P<0.05
Self-employed	27.4	21.7		
Housewife	17.8	15.7		
No occupation	5.9	6.5		
**Participate in saving group (%)**	69.8	48.5	χ^2^ = 41.3	P<0.001
**Run own small business**	37.6	19	χ^2^ = 36.2	P<0.001
**Household size (Mean)**	5	4.3	t = 5.15	P<0.001
**HHD monthly income in Birr (Mean)**	478	416	t = 3.72	P<0.001
**Currently growing garden vegetables or fruits**	90.4	13.6	X2 = 52.6	P<0.01

### Frequency of FV consumption

**Fig 1:**
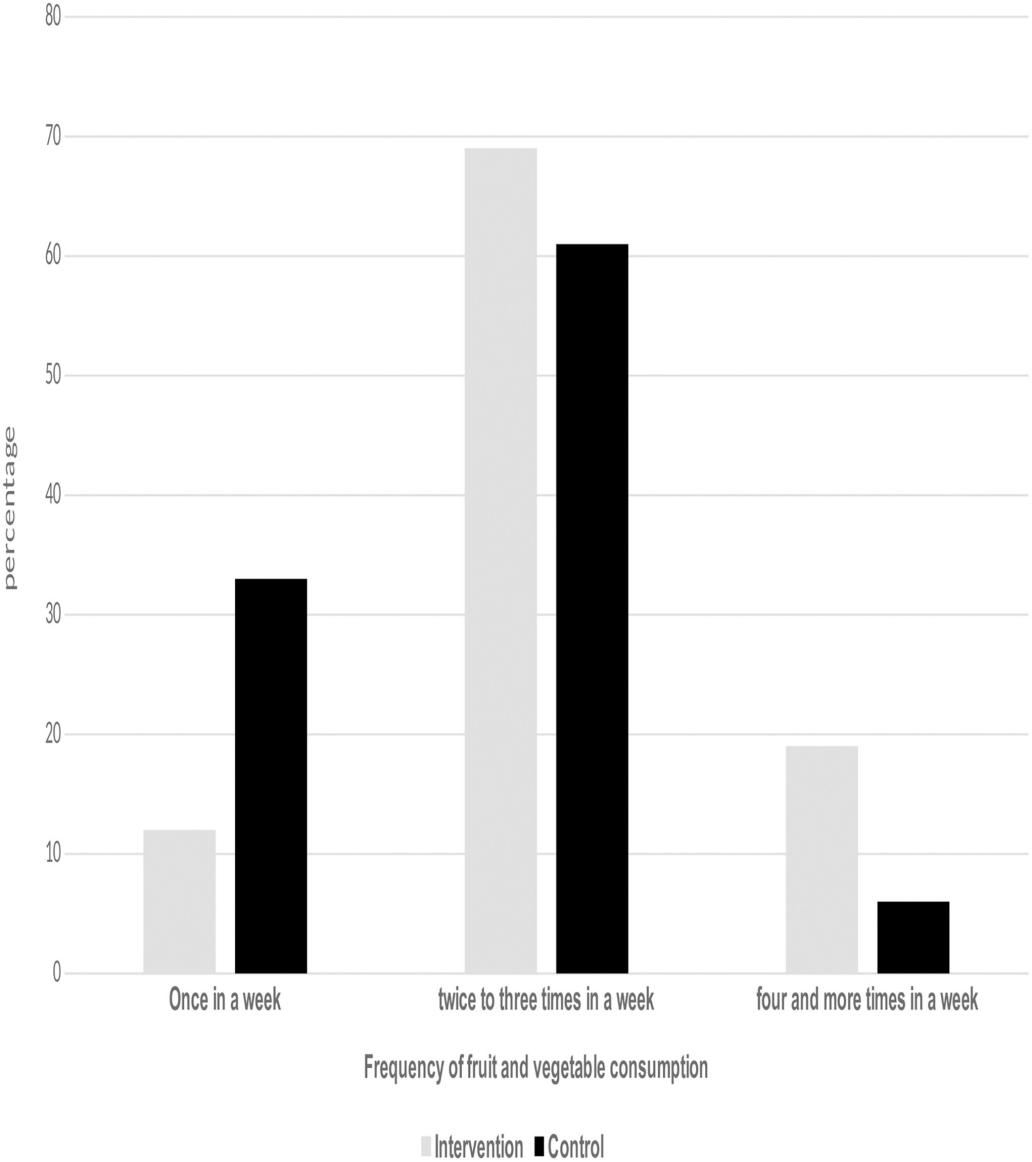
Frequency of vegetables and fruits consumption.

Fig 1 shows the frequency of one-week of FV consumption by intervention and control groups. Out of the total 759 participants (404 intervention, 355 control), 88.4% of the intervention groups reported consuming FV at least twice in a week compared with 67.3% participants from control group that reported consuming FV twice and more times in a week (chi-square = 49.6, P<0.01). About 19% of households in the intervention group consumed FV four or more times in a week compared with 6% in the control group (chi-square: 66.7, P<0.01).

### Diversity of FV consumption

**Fig 2:**
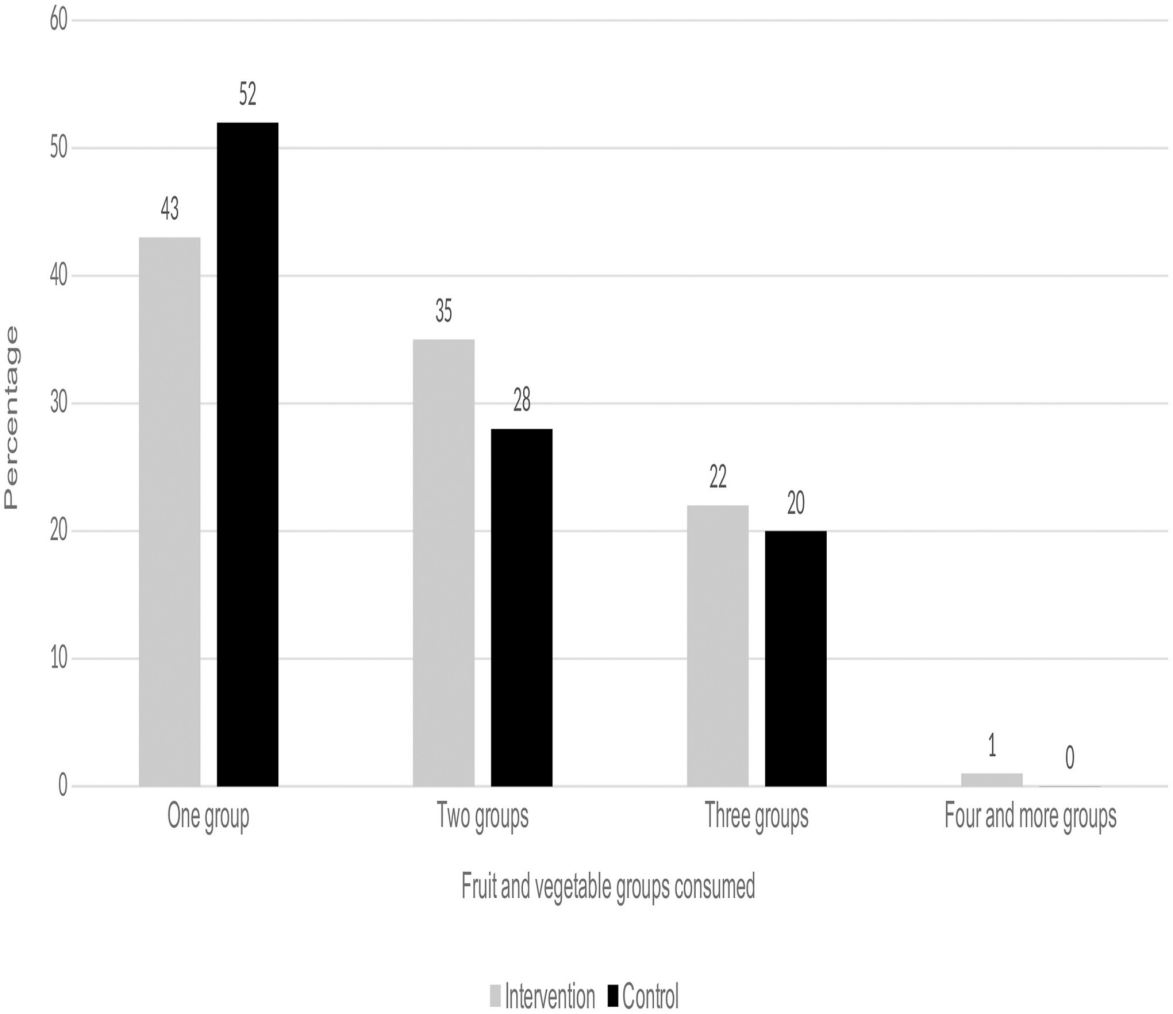
Diversity of 24 hour vegetable and fruit consumption among intervention and control groups.

Fig 2 shows the 24 hour FV groups consumption by intervention and control groups. Results show that there was a significant difference between the intervention and control groups in the diversity of the 24 hour FV consumption. Out of the total 626 participants (354 intervention, 272 control), 57% of participants in the intervention group reported consuming more than one group of vegetable (had a diversity of FV consumption) compared with 48% participants in the control groups (chi-square = 5.304, p<0.05).

Table 2 Summarizes the specific groups of FV consumed in the previous 24 hour. The percentage of households that reported consuming vitamin A rich vegetable is almost equal to the percentage of HH in the intervention group. 98.3% of study participants in the intervention group reported consuming dark green vegetables compared with 96.3% of HH in control group (P = 0.757). 42% of study participants in the intervention group have reported consuming vitamin A rich fruits compared with 30.5% of HH in control group (p< 0.005). 7.1% of study participants in the intervention group have reported consuming any other vegetables compared with 30.5% of HH in control group (p = 0.754).

**Table 2:** Summary of the 24 hour household vegetable and fruit consumption by intervention and control groups.

The 24 hour household vegetable and fruit consumption	Intervention group	Control groups	Percentage difference	Chi-Square test	P-Value
	Frequency/ %	Frequency/ %			
Consumed at least 2 groups of fruit and vegetable.	202 (57)	130 (48)	9	5.34	P<0.05
Consumed vitamin A rich vegetables and tubers (group I).	113 (32)	90 (33)	1	0.096	0.757
Consumed dark green leafy vegetables. (group III).	348 (98.3)	262 (96.3)	2	2.425	0.119
Consumed other vegetables (group IV).	25 (7.1)	21 (7.7)	0.6	0.098	0.754
Consumed vitamin A rich fruits. (group II).	149 (42.0)	83 (30.5)	11.5	8.836	P<0.005

### Access to FV from own garden

**Fig 3:**
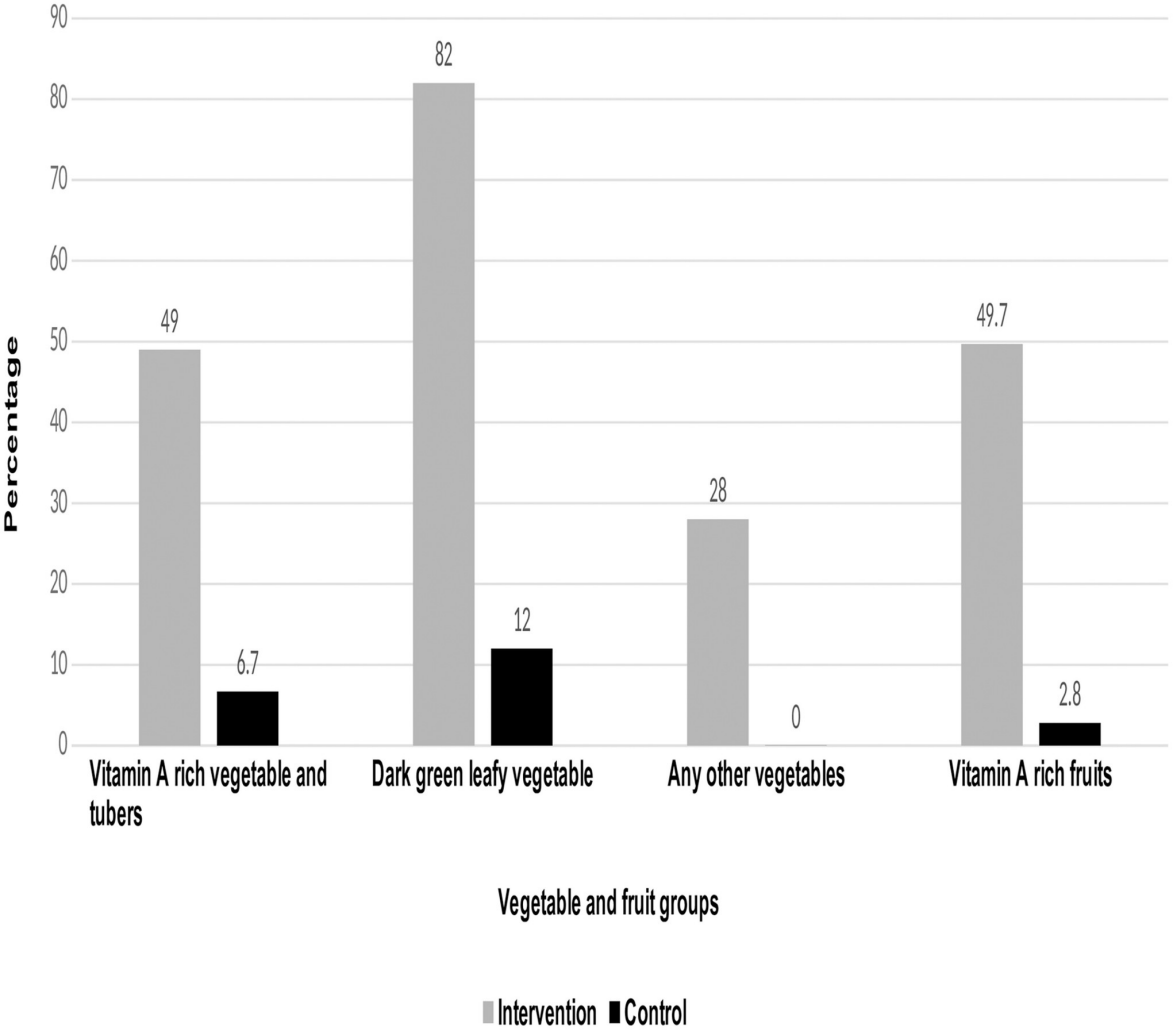
Percentage of participants with the source of vegetables and fruits and vegetables from own garden.

A significant higher proportion of participants from intervention groups (65.8%) compared with control groups (7.5%) reported getting FV from their own garden for their one-week consumption (chi-square2 = 282, p<0.001). Consistently, the source of 24 hour FV consumption shows that fourty nine percent of participants in the intervention reported getting vitamin A rich plants from their garden compared with 6.7 percent participates in the control group (chi-square = 43.5, p<0.001). Eighty two percent of intervention participants reported that they have got dark green vegetables from their garden compared with 12 percent of participates in the control group that reported getting dark green vegetables from their garden (chi-square = 291, p<0.001). Fifty percent of intervention participants reported that they have got the vitamin A rich fruits from their garden compared with 5 percent of participates in the control group that reported getting the vitamin A rich fruits from their garden (chi-square2 = 49.9, p<0.001). Twenty eight percent of intervention participants reported that they got any other vegetables from their garden compared with 0% participates in the control group that reported getting any other vegetables from their garden (chi-square = 7.0, p<0.001) (Fig 3).

### Attitude towards eating fruit and vegetable and future intention to practice home gardening

Table 3 Shows perceived importance to include FV in daily meals and the likelihood of practicing home gardening in the future by intervention and control groups. Sixty six percent of participants in the intervention group and fifty four percent of participants in the control group reported “highly important” to include FV in every day diet (chi-square = 13.2, p<0.001). Regarding practicing home gardening in the future, 58% of the control group and 88% of the intervention group reported high likely’ to practice home gardening in the future (chi-square = 89.7, p<0.001).

**Table 3:** Perceived highly importance to include fruit and vegetables in every day meal and the likelihood of practicing home gardening in the future by intervention and control groups.

Perceived importance on vegetables and intention to future gardening	Percentage of intervention groups (N = 414)	Percentage of control groups (N = 443)	Chi-square	P-value
**Percieved imporrtance to vegetables in every day meal**				
Not much important	0	0.5	13.2	p<0.001
Important	34	45		
Highly important	66	54.5		
**The likelihood of practicing home gardening in the future**	**(N = 414)**	**(N = 376)**		
Highly likely	88%	58%	98.7	p<0.001
Likely	12%	32%		
Not likely	0%	9.50%		

## Discussion

This study was conducted among caregivers of highly vulnerable children in seven *woreda* of Ethiopia and examined whether the groups that implemented permagarden have higher FV intake compared with their control. Results show that intervention groups have higher frequency and diversity of FV intake, and they have higher intention to practice gardening in the future compared with control groups.

After controlling for family income using PSM, results of this study showed that permagarden intervention groups consumed FV more times per week than control groups. A significant higher percentage of participants in the intervention group reported getting their family consumption of FV form their own garden compared with controls. Studies reported that availability and accessibility of FV in the home was positively associated with increased consumption [37]. Similar to this study, other researchers have shown that home gardens have a high impact in increasing access to fresh FV [20,38]. Thus, home gardens can contribute to improving access to food to vulnerable households and their families [39,40,41].

Even if there is a significant difference in both 24 hour FV consumption and weekly FV consumption between intervention group and control group, FV intake in both groups is still low compared to the WHO standard, which is about 400 grams per day [3,4]. Out of the total participants, 42% of intervention and 52% of the control groups consumed only one type of vegetable in 24 hours. Only 19% of intervention households and 6% of control households consumed FV more than 3 times in a week. Studies reported that home-garden interventions are most effective when combined with promotional and educational interventions [42]. Based on the result from this study, and other studies, we suggest that home garden programs should integrate nutrition education [17,43]. In addition to assessing the impact of permagarden on fruit and vegetable intake, it would be important to identify the determinants of FV intake, which was not directly addressed in this study.

This study shows that the participants’ perception to include FV in in everyday meals was higher in intervention group compared to control. This finding suggests that participation in the permagarden intervention might have influenced participants to develop positive attitude to FV consumption. Consistent to this finding, other studies reported that participating in gardening increased preferences for vegetables [44,45].

Several studies reported the impact of home gardens in increasing FV consumption and the findings have consistently shown that it is a very effective strategy [39,40,41]. However, most studies were conducted in developed countries [17,46,47,48], most of them infer a large space garden implemented in places like schools [20,44], among them there are fewer studies that targeted highly vulnerable societies [17,20,44,46,47,48]. Since evidence has shown that the poorest people had the highest prevalence of low FV consumption [46,48,49]. This makes the program model a viable option in urban area to address the food and nutrition gap among vulnerable societies of least developed countries [50]. Since the current findings are based on comparison of intervention and control groups at study end, evidence from future randomized intervention studies with baseline data are likely to generate stronger conclusion with programmatic implications.

### Strengths and limitations

A strength of the current pilot study was its ability to control confounding factors through PSM. Several study limitations may affect the interpretation of these results. The study was not a randomized controlled trial. Based on a one-time post-intervention data in this study, it might be difficult to establish causal relationship between the exposure and observed outcomes. We have estimated FV consumption based on the participant’s self-reported responses that might reduce the level of confidence we can put on the strength of the intervention effect.

### Conclusions

The observed higher FV intake among permagarden intervention group compared to control group suggest that nutrition and health programs need to promote permagarden as a means to improve FV intake among vulnerable societies in least and middle developed countries.

## Supporting information

S1 File File_Permagarden1. (SAV)

## Abbreviations

CC: Community Committee
CCC: Community Care coalition
CF: Community Facilitators
CSI: Child Support Index
CSO: Civil Society Organization
FHI 360: Family Health International 360
FV: Fruits and Vegetables
GOE: Government of Ethiopia
HVC: Highly Vulnerable Children
IEC: Independent or Institutional Ethics Committee
IRB: Institutional Review Board
NIH: National Institutes of Health
OVC: Orphan and Vulnerable Children
PHSC: Protection of Human Subjects Committee
SD: Standard Deviation
TOT: Training of Trainers
UNICEF: United Nations Children’s Fund
USAID: United States Agency for international Development
USD: US Dollar
WHO: World Health Organization
